# Genetic proof of herpesviruses CMV, EBV and HHV-6 in intestinal biopsies from patients with inflammatory bowel diseases: prevalence and disease characteristics

**DOI:** 10.1093/crocol/otag009

**Published:** 2026-01-27

**Authors:** Chengcheng Christine Zhang, Sarah Klemm, Paul Schnitzler, Annika Gauss

**Affiliations:** Department of Gastroenterology, University Hospital Heidelberg, Heidelberg 69120, Germany; Department of Infectious Diseases, Virology, University Hospital Heidelberg, Heidelberg 69120, Germany; Department of Infectious Diseases, Virology, University Hospital Heidelberg, Heidelberg 69120, Germany; Department of Gastroenterology, University Hospital Heidelberg, Heidelberg 69120, Germany

**Keywords:** CMV, EBV, HHV-6, inflammatory bowel disease, ulcerative colitis, Crohn’s disease

## Abstract

**Background:**

While the clinical relevance of cytomegalovirus (CMV) for inflammatory bowel disease (IBD) patients remains controversial, the role of related Epstein-Barr virus (EBV) and human herpesvirus 6 (HHV-6) is unknown. The aim of this retrospective study was to determine the prevalence of CMV, EBV, and HHV-6 DNA in the intestinal mucosa of IBD patients, and to identify potential risk factors and associations with disease characteristics.

**Methods:**

Any IBD patient who underwent endoscopy with mucosal biopsy for virologic assessment at Heidelberg University Hospital from December 2018 to September 2020 was eligible. DNA of CMV, EBV, and HHV-6 was determined by polymerase chain reaction.

**Results:**

A total of 230 patients were included (123 male, median age 37 years). Ulcerative colitis was diagnosed in 146 patients (63.5%), Crohn’s disease in 78 patients (33.9%), and unclassified IBD in 6 patients (2.6%). The prevalence of CMV was 6.1% (*n* = 14/230), 25.7% for EBV (*n* = 59/230), and 8.7% for HHV-6 (*n* = 20/230). Detection of EBV DNA was associated with longer hospitalization (*P *= 0.001), steroid treatment (*P *= 0.004), higher CRP levels (*P *= 0.04), and higher disease activity (*P *= 0.02). Histological evidence of inflammation (*P *= 0.03) and ulcers were also detected significantly more often in EBV-positive patients (*P *= 0.001). JAK-inhibitor treatment was associated with CMV infection, while HHV-6-positive patients were treated more frequently with azathioprine/6-mercapotopurine or steroids.

**Conclusions:**

The study reveals that the prevalence of EBV in the intestinal mucosa is high in patients with IBD and correlates with disease activity and severity, while HHV-6 is associated with specific immunosuppressive therapy.

## Graphical Abstract

**Graphical Abstract:**
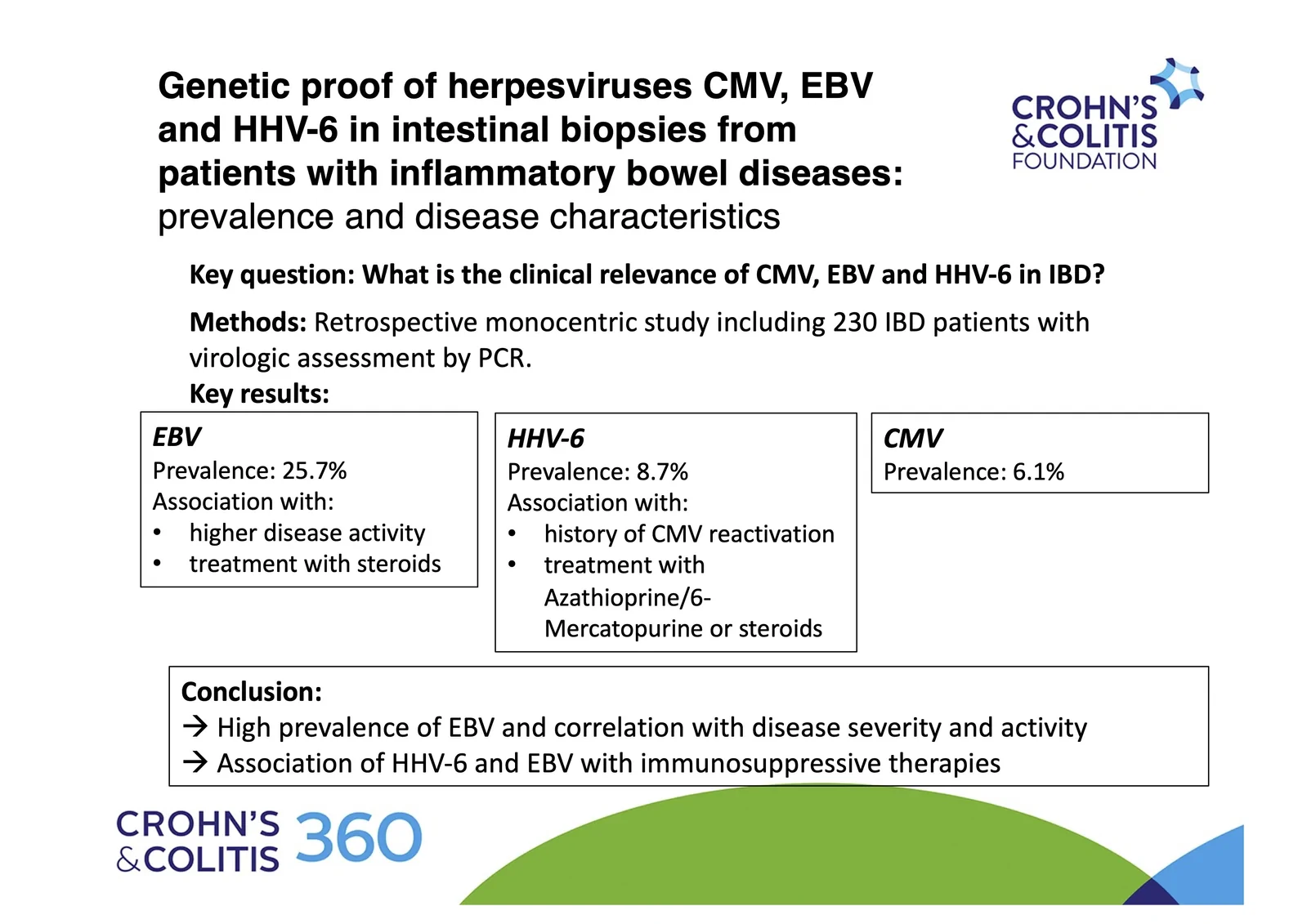

## Introduction

To date, the etiology and pathogenetic mechanisms of inflammatory bowel disease (IBD) have yet to be unraveled. Despite advances in treatment options and medications, the affected patients’ quality of life is significantly reduced. Therapeutic strategies have shifted over the last decades to earlier and more aggressive treatment in order to achieve mucosal healing and prevent disease progression.^1^ Hence, a substantial number of patients are treated with immunosuppressive medications or biologicals, for example, steroids, thiopurines, tumor necrosis factor α (TNFα) inhibitors, integrin inhibitors, interleukin (IL) (12/)23-inhibitors, Janus kinase (JAK) inhibitors, calcineurin inhibitors, methotrexate, and sphingosin-1-phosphate receptor (S1PR) modulators. These treatments may lead to an increased risk of gastrointestinal infections. Furthermore, IBD activity compromises the functional integrity of the intestinal mucosal barrier, making the patients more vulnerable to infections.

An association between cytomegalovirus (CMV) infection in the intestinal mucosa and ulcerative colitis (UC) was first reported in 1961.^2^ Since then, several studies have shown that the intestinal reactivation of CMV is associated with severe disease activity in IBD patients.^3–5^ In particular, patients with refractory UC harbored higher mucosal concentrations of CMV DNA compared to UC patients with uncomplicated disease phenotypes and patients with Crohn’s disease (CD).^6^ As suggested by the European Crohn’s and Colitis Organization (ECCO) guideline 2021,^7^ a number of options can be used to diagnose intestinal CMV infection: the detection of viral DNA by polymerase chain reaction (PCR) testing from blood samples or tissue biopsies, the combined histopathology and immunohistochemical detection of CMV-infected cells in mucosal tissue of the large intestine, and positive CMV antibody/antigen detection in the blood. Of these methods, the most sensitive method is the detection of viral DNA by PCR. In cases of assumed clinical relevance, patients with intestinal CMV reactivation should be treated with (val)ganciclovir.^7^

In addition to CMV, other members of the herpesvirus family such as HHV-6 and Epstein-Barr virus (EBV) can be detected in intestinal tissue and stool samples from IBD patients. For UC patients, the reported prevalence of CMV and EBV DNA in stool samples was 36.6%, and for HHV-6 11.3%.^8^ Concomitant detection of HHV-6 along with CMV or EBV DNA in colonic tissue samples was more common in patients with UC compared to controls (76% vs. 29%), and patients with active UC were at higher risk for overlapping virus detection.^9^ The frequency of CMV detection in intestinal mucosa ranges from 10% to 36%,^3^^,^^10–12^ while that of EBV seems to be higher, ranging from 16% to 79.4%.^13–15^

Despite these correlations, the pathogenetic and clinical relevance of EBV, HHV-6, and CMV infection in the intestinal mucosa of IBD patients remains elusive. Therefore, the aim of this study was to determine the prevalence of CMV, EBV, and HHV-6 DNA in intestinal mucosal tissue samples from patients with IBD, and to investigate associations between these pathogens and clinical disease course, disease activity, and endoscopic/histological findings. To the best of our knowledge, this is the first study including HHV-6 analysis and comparing all 3 herpesviruses in a comprehensive approach.

## Methods

### Study design and study population

The aim of this uncontrolled, monocentric, retrospective study was to determine the prevalence of the genetic material of herpesviruses CMV, EBV, and HHV-6 in the intestinal mucosa of patients with IBD, and to investigate potential associations of these pathogens with disease activity and disease course. Further parameters to be considered were: concurrent medical treatment, response to steroid therapy, and need for surgical treatment until the end of follow-up.

IBD patients were eligible for inclusion in the study if they had mucosal tissue samples taken from the sigmoid for routine virologic assessment of CMV DNA at the interdisciplinary center of endoscopy at Heidelberg University Hospital from December 1, 2018 until September 30, 2020. The treating physician decided whether to take biopsies for CMV detection. The diagnosis of IBD (ie, UC, CD, or unclassified IBD [IBD-U]) was made according to ECCO criteria.^16^ Study cohort exclusion criteria were patients younger than 18 years of age, and instances where there was insufficient sample material for further virologic analyses. The processed biopsy specimens were routinely stored at the Department of Virology of Heidelberg University Hospital.

Clinical, demographic, endoscopic, and laboratory data were collected from fully electronic medical records. Data of the identified patients were entered into a Microsoft Excel spreadsheet. They included demographic data such as age, sex, and body mass index, as well as details on disease course and therapy (including, but not limited to, disease duration, concomitant medications, extent of disease, clinical symptoms, endoscopic features, hospitalizations, necessity of colectomy, and history of CMV reactivation which was defined as detection of CMV replication in blood or CMV-positive mucosal samples), and laboratory parameters. The laboratory parameters included were plasma C-reactive protein (CRP), hemoglobin, and blood leukocyte and platelet counts. The Montreal classification was used to categorize disease phenotypes.^17^ Clinical activity was assessed using the SCCAI (simple clinical colitis activity index) for UC^18^ and the Harvey-Bradshaw Index (HBI) for CD.^19^ The Mayo scoring system was used to evaluate endoscopic activity for UC patients.^20^ For CD patients, the simple endoscopic score for Crohn’s disease (SES-CD) scoring system was used if patients had undergone complete colonoscopy.^21^ Disease activity parameters and endoscopy scores were calculated retrospectively from the patient records.

### Virologic analyses

Detection of DNA from CMV, EBV, and HHV-6 (both HHV-6A and HHV-6B) in biopsies was based on qualitative PCR technique. DNA was extracted from biopsies using the QIAamp DNA Mini Kit (Qiagen). Amplification and detection of viral DNA was conducted with RealStar real-time PCR kits for CMV, EBV, and HHV-6 (altona Diagnostics) on a LightCycler 480 instrument (Roche Diagnostics). According to manufacturer’s instructions, the thresholds used to determine a positive DNA test result in the qualitative PCR assays were 100 IU/mL for CMV, 1000 IU/mL for EBV, and 1000 copies/mL for HHV-6.

### Statistical analysis

Statistical analyses were performed using SPSS Statisticsv29 (IBM Corporation). Descriptive statistics were calculated as percentages for discrete variables and presented as medians with ranges for continuous variables. If the requirement of normal distribution was met, the *t*-test was performed. If this condition was not met and the data were ordinally scaled, the Mann–Whitney *U*-test was used for comparison. To compare categorical variables, the Chi-squared test was used. Factors associated with the presence of CMV, EBV, and HHV-6 DNA in the intestinal mucosa of patients with IBD were analyzed using a univariate logistic regression model. Covariates that showed significant results in the univariate analysis were further analyzed in multivariate analysis. A *P *< 0.05 was considered statistically significant.

### Ethical considerations

Data acquisition and evaluation were approved by the local ethics committee (S-670/2020) and conformed to the ethical guidelines of the Declaration of Helsinki, as reflected in an *a priori* approval by the institution’s human research review committee. For retrospective determination of viral DNA in existing intestinal biopsies and access to data from the hospital information system, the collection of informed consent from the patients was waivered by the authorities, as the research purpose would thus only have been achievable with a disproportionately high amount of work.

## Results

### Baseline characteristics of the included patients

A total of 230 patients were included (123 male, median age 37 years) with a median follow-up of 12 months (range 0-33 months). UC was diagnosed in 146 patients (*n* = 146/230; 63.5%), CD in 78 patients (*n* = 78/230; 33.9%), and IBD-U in 6 patients (*n* = 6/230; 2.6%). In total, 55 patients (*n* = 55/230; 22.2%) were on anti-TNFα treatment at the time of biopsy sampling, 36 (*n* = 36/230; 15.7%) had anti-IL 12/23 therapy, 24 (*n* = 24/230; 10.4%) were treated with integrin inhibitors, 21 (*n* = 21/230; 9.1%) had calcineurin inhibitor treatment, 26 (*n* = 26/230; 11.3%) were on azathioprine or 6-mercaptopurine therapy, 17 (*n* = 17/230; 7.4%) received JAK-inhibitor treatment, 1 (*n* = 1/230; 0.4%) had methotrexate therapy, and 88 (*n* = 88/230; 38.3%) were treated with steroids (69 [30%] orally and 19 [8.3%] intravenously). A total of 96 patients (*n* = 96/230; 41.7%) received combined immunosuppressive treatment at the time of biopsy sampling. The median disease duration until biopsy was 8 years. A total of 58 patients (*n* = 58/230; 25.2%) had undergone bowel resection(s) for their IBD, and 32 (*n* = 32/230; 13.9%) had a documented history of intestinal CMV reactivation. Regarding clinical disease activity, the median SCCAI for patients with UC and IBD-U was 6, and for patients with CD, the median HBI was 6. The median Mayo score for evaluating endoscopic disease activity for patients with UC was 2, while for patients with CD, the median SES-CD score was 7. CMV DNA was found in the intestinal mucosa of 14 patients (*n* = 14/230; 6.1%), while EBV DNA was detected in 59 patients (*n* = 59/230; 25.7%), and HHV-6 positive samples were found in 20 patients (*n* = 20/230; 8.7%). At the time of biopsy, 132 of the patients (*n* = 132/230; 57.4%) were hospitalized. The colectomy rate within the study cohort was 2.2% (*n* = 5/230), with EBV DNA detected in 4 colectomy patients and HHV-6 DNA in 1 patient. Among these colectomy patients, there were no instances of concurrent viral DNA positivity. The relapse rate was 17.8% (*n* = 41/230). Out of these patients, 9 tested positive for viral DNA (8 patients with EBV and 1 patient with HHV-6). The 30-day mortality rate was 0%. Detailed baseline characteristics are shown in Table 1.

**Table 1:** Patient characteristics.

	IBD	UC	CD	IBD-U
	*n* = 230	*n* = 146	*n* = 78	*n* = 6
**Baseline characteristics**				
**Male, *n* (%)**	123 (53.5)	85 (58.2)	37 (47.4)	1 (16.7)
**Age at diagnosis of IBD (years), median (range)**	25 (4-78)	25 (4-78)	25 (7-76)	17 (26-38)
**Age at biopsy (years), median (range)**	37 (18-82)	36 (18-80)	42 (18-82)	33 (25-59)
**Disease duration at biopsy (years), median (range)**	8 (0-51)	6 (0-51)	9 (0-42)	4 (0-31)
**History of CMV reactivation, *n* (%)**	32 (13.9)	27 (18.5)	4 (5.1)	1 (16.7)
**History of bowel resection(s), *n* (%)**	58 (25.2)	22 (15.1)	36 (46.2)	0 (0)
**In-patient stay at time of biopsy, *n* (%)**	132 (57.4)	51 (39.4)	22 (28.2)	1 (16.7)
**Duration of in-patient stay (days), mean** **±** **SD**	15 ± 17	17 ± 20	11 ± 8	8 ± 0
**Relapse rate, *n* (%)**	41 (17.8)	33 (22.6)	7 (9)	1 (16.7)
**Colectomy, *n* (%)**	5 (2.2)	5 (3.4)	0 (0)	0 (0)
**30-day mortality, *n* (%)**	0 (0)	0 (0)	0 (0)	0 (0)
**Medical treatment**				
**Anti-TNFα treatment at biopsy, *n* (%)**	51 (22.2)	28 (19.2)	21 (26.9)	2 (33.3)
**Anti-interleukin 12/23 treatment at biopsy, *n* (%)**	36 (15.7)	16 (11)	19 (24.4)	1 (16.7)
**Anti-integrin treatment at biopsy, *n* (%)**	24 (10.4)	21 (14.4)	3 (3.8)	0 (0)
**Calcineurin inhibitor treatment at biopsy, *n* (%)**	21 (9.1)	19 (13)	2 (2.6)	0 (0)
**Azathioprine/6-Mercatopurine treatment at biopsy, *n* (%)**	26 (11.3)	13 (8.9)	11 (14.1)	2 (33.3)
**Methotrexate treatment at biopsy, *n* (%)**	1 (0.4)	0 (0)	1 (1.3)	0 (0)
**JAK-inhibitor treatment at biopsy, *n* (%)**	17 (7.4)	13 (8.9)	3 (3.8)	1 (16.7)
**Steroid treatment (intravenous) at biopsy, *n* (%)**	19 (8.3)	17 (11.6)	2 (2.6)	0 (0)
**Steroid treatment (oral) at biopsy, *n* (%)**	69 (30)	45 (30.8)	22 (28.2)	2 (33.3)
**Combined treatment at biopsy, *n* (%)**	96 (41.7)	71 (48.6)	22 (28.2)	3 (50)
**Montreal classification**				
**Location, *n* (E1: E2: E3) (UC and IBD-U)**		6:41:91		
**Location, *n* (L1: L2: L3: L4) (CD)**			14:23:42:9	
**Behavior, *n* (B1: B2: B3) (CD)**			30:28:29	
**Perianal, *n* (%) (CD)**			25 (32.1)	
**Clinical disease activity**				
**SCCAI at biopsy, median (range) (UC and IBD-U, *n* = 137)**		6 (0-14)		
**HBI at biopsy, median (range) (CD, *n* = 76)**			6 (0-36)	
**Laboratory parameters**				
**CRP (mg/L), mean** **±** **SD**	23 ± 37	24 ± 38	19 ± 27	43 ± 81
**Calprotectin (µg/g), mean** **±** **SD**	979 ± 711	1033 ± 721	871 ± 681	1800 ± 0
**Endoscopic disease activity**				
**Mayo score, median (range) (UC)**		2 (0-3)		
**SES-CD score, median (range) (CD)**			7 (0-27)	
**Endoscopic findings**				
**Presence of ulcers, *n* (%)**	113 (49.1)	69 (47.3)	42 (53.8)	2 (33.3)
**Deep ulcers, *n* (%)**	27 (11.7)	14 (9.5)	12 (15.4)	1 (16.7)
**Histological findings**				
**Acute inflammation, *n* (%)**	181 (78.7)	118 (80.8)	59 (75.6)	4 (66.7)
**Chronic inflammation, *n* (%)**	170 (73.9)	112 (76.7)	53 (67.9)	5 (83.3)
**Virologic evaluation**				
**Positive CMV PCR, *n* (%)**	14 (6.1)	8 (5.5)	5 (6.4)	1 (16.7)
**Positive EBV PCR, *n* (%)**	59 (25.7)	44 (30.1)	14 (17.9)	1 (16.7)
**Positive HHV-6 PCR, *n* (%)**	20 (8.7)	14 (9.6)	6 (7.7)	0 (0)

Abbreviations: CD: Crohn’s disease; CMV: cytomegalovirus; EBV: Epstein-Barr virus; HHV-6: human herpesvirus-6; IBD-U: IBD unclassified; HBI: Harvey-Bradshaw index; IBD: inflammatory bowel disease; JAK: Janus kinase; PCR, polymerase chain reaction; SCCAI simple clinical colitis activity index; SES-CD: simple endoscopic score for Crohn′s disease; TNFα: tumor necrosis factor alpha; UC: ulcerative colitis.

### Cytomegalovirus

CMV DNA was found in 14 patients (*n* = 14/230; 6.1%). Among these 14 patients (6 male, median age 41 years), UC was diagnosed in 8 patients, CD in 5 patients, and IBD-U in 1 patient. JAK-inhibitor treatment was used significantly more often in CMV-positive patients compared to patients with negative test results (*P *= 0.04). Furthermore, both the SCCAI for patients with UC and IBD-U and the Mayo score for determining endoscopic disease activity were significantly higher for CMV-negative patients, compared to patients with positive test results (*P *= 0.01 and *P *= 0.005, respectively). Regarding the other medical therapies and characteristics, there was no significant difference among the subgroups. EBV was detected concurrently in 3 CMV-positive patients (*n* = 3; 21.4%), while none of them had concurrent positive HHV-6 results.

Among the 32 patients with a documented history of intestinal CMV reactivation in the whole study cohort, 2 patients (*n* = 2/32; 6.25%) were tested positive again for CMV within the time frame of the current investigation.

Out of the 14 patients who tested positive for CMV, 8 patients discontinued or changed immunosuppressive therapy (*n* = 8/14; 57%), 7 patients showed clinical improvement after change of therapy (*n* = 7/14; 50%), and 1 patient (*n* = 1/14; 7.1%) with therapy-refractory colitis was advised to undergo colectomy, but declined the operation until the last follow-up. No patient who tested positive for CMV underwent colectomy during follow-up. Detailed characteristics of patients with CMV-positive and -negative PCR are shown in Table 2.

**Table 2:** Comparison of characteristics between the subgroups of patients with positive CMV PCR and negative CMV PCR.

	CMV PCR-positive	CMV PCR-negative	*P*-value
	*n* = 14/230	*n* = 216/230	
**Variable**			
**Male, *n* (%)**	6 (42.9)	115 (53.2)	0.78
**IBD specification, *n* (%)**			
**UC, *n* (%)**	8 (57.1)	138 (63.9)	0.52
**CD, *n* (%)**	5 (35.7)	73 (33.8)	0.31
**IBD-U, *n* (%)**	1 (7.1)	5 (2.3)	0.27
**Age at diagnosis of IBD (years), median (range)**	26 (15-64)	25 (4-78)	0.79
**Age at biopsy (years), median (range)**	41 (23-75)	37 (18-82)	0.34
**Disease duration at biopsy (years), median (range)**	10 (0-44)	7 (0-51)	0.26
**History of CMV reactivation, *n* (%)**	2 (14.3)	30 (13.9)	0.98
**History of bowel resection(s), *n* (%)**	5 (35.7)	53 (24.5)	0.36
**In-patient stay at time of biopsy, *n* (%)**	3 (21.4)	71 (32.9)	0.37
**Duration of in-patient stay (days), mean** **±** **SD**	4 ± 2	15 ± 17	0.3
**Medical treatment**			
**Anti-TNFα treatment at biopsy, *n* (%)**	3 (21.4)	48 (22.2)	0.94
**Anti-interleukin 12/23 treatment at biopsy, *n* (%)**	3 (21.4)	33 (15.3)	0.55
**Anti-integrin treatment at biopsy, *n* (%)**	2 (14.3)	22 (10.2)	0.63
**Calcineurin inhibitor treatment at biopsy, *n* (%)**	1 (7.1)	20 (9.3)	0.78
**Azathioprine/6-Mercatopurine treatment at biopsy, *n* (%)**	2 (14.3)	24 (11.1)	0.72
**Methotrexate treatment at biopsy, *n* (%)**	0 (0)	1 (0.5)	0.79
**JAK-inhibitor treatment at biopsy, *n* (%)**	3 (21.4)	14 (6.5)	**0.04**
**Steroid treatment (intravenous) at biopsy, *n* (%)**	1 (7.1)	18 (8.3)	0.87
**Steroid treatment (oral) at biopsy, *n* (%)**	5 (35.7)	64 (29.6)	0.64
**Combined treatment at biopsy, *n* (%)**	7 (50)	89 (41.2)	0.53
**Montreal classification of UC and IBD-U:**			
**E1**	1 (12.5)	5 (3.6)	0.27
**E2**	2 (25)	39 (28.3)	0.72
**E3**	5 (62.5)	86 (62.3)	0.77
**Montreal classification of CD:**			
**L1**	1 (20)	13 (17.8)	0.33
**L2**	2 (40)	21 (28.8)	0.71
**L3**	2 (40)	40 (54.8)	0.69
**L4**	0 (0)	9 (12.3)	0.44
**B1**	2 (40)	28 (38.4)	0.14
**B2**	0 (0)	28 (38.4)	0.15
**B3**	3 (60)	26 (35.6)	0.31
**Perianal disease**	3 (60)	22 (30.1)	0.19
**Clinical disease activity**			
**SCCAI at biopsy, median (range) (UC and IBD-U, *n* = 137)**	2.5 (0-6)	6 (0-14)	**0.01**
**HBI at biopsy, median (range) (CD, *n* = 76)**	6 (0-9)	6 (0-36)	0.44
**Laboratory parameters**			
**CRP (mg/L), mean** **±** **SD**	5 ± 5	24 ± 38	**0.007**
**Calprotectin (µg/g), mean** **±** **SD**	1047 ± 803	974 ± 710	0.46
**Endoscopic disease activity**			
**Mayo score, median (range) (UC)**	1 (0-2)	2 (0-3)	**0.005**
**SES-CD score, median (range) (CD)**	6.5 (5-8)	7 (0-27)	0.61
**Endoscopic findings**			
**Presence of ulcers, *n* (%)**	6 (42.9)	107 (49.5)	0.61
**Deep ulcers, *n* (%)**	1 (7.1)	26 (12.3)	0.57
**Histological findings**			
**Acute inflammation, *n* (%)**	9 (64.3)	172 (79.6)	0.31
**Chronic inflammation, *n* (%)**	8 (57.1)	162 (75)	0.24
**Simultaneous viral DNA detection**			
**Positive EBV PCR**	3 (21.4)		
**Positive HHV-6 PCR**	0 (0)		

Abbreviations: CD: Crohn’s disease; CMV: cytomegalovirus; EBV: Epstein-Barr virus; HHV-6: human herpesvirus-6; IBD-U: IBD unclassified; HBI: Harvey-Bradshaw Index; IBD: inflammatory bowel disease; JAK: Janus kinase; PCR, polymerase chain reaction; SCCAI simple clinical colitis activity index; SES-CD: simple endoscopic score for Crohn′s disease; TNFα: tumor necrosis factor alpha; UC: ulcerative colitis.

Bold values indicate significant *P*-values (<0.05).

### Epstein-Barr virus

EBV DNA was found in 59 patients (*n* = 59/230; 25.7%). Out of these 59 patients (35 male, median age 36 years), UC was diagnosed in 44 patients (*n* = 44/59; 74.6%), CD in 14 patients (*n* = 14/59; 23.7%), and IBD-U in 1 patient (*n* = 1/59; 1.7%). Patients who tested positive for EBV DNA were significantly more likely to be hospitalized at the time of biopsy compared to patients with negative PCR results (*P *= 0.001), and the duration of hospitalization for EBV-positive patients was significantly longer, with a mean stay of 19 days, compared to 12 days for EBV-negative patients (*P *= 0.001). Furthermore, EBV-positive patients required significantly more intravenous steroid treatment than EBV-negative patients (*P *= 0.004). Regarding the laboratory parameters, patients testing positive for EBV had significantly higher mean CRP levels (*P *= 0.04). Moreover, it was found that both the SCCAI for patients with UC and IBD-U and the Mayo score for determining endoscopic disease activity were significantly higher for EBV-positive patients than for EBV-negative patients (*P *= 0.02 and *P *= 0.003, respectively).

CMV was detected concurrently in 3 EBV-positive patients (*n* = 3; 5.1%), while 6 patients tested positive for both EBV and HHV-6 DNA concurrently (*n* = 6; 10.2%).

Immunosuppressive therapy was discontinued in 3 out of 59 patients after biopsy (*n* = 3/59; 5.1%). A total of 4 EBV-positive patients underwent colectomy due to therapy refractory colitis during the course of their disease (*n* = 4/59; 6.8%), with 3 of those discontinuing immunosuppressive therapy due to planned surgery.

Detailed characteristics of patients with EBV-positive and -negative PCR test results are shown in Table 3.

**Table 3:** Comparison of characteristics between the subgroups of patients with positive EBV PCR and negative EBV PCR.

	EBV PCR-positive	EBV PCR-negative	*P*-value
	*n* = 59/230	*n* = 171/230	
**Variable**			
**Male, *n* (%)**	35 (59.3)	88 (51.5)	0.3
**IBD specification, *n* (%)**			
**UC, *n* (%)**	44 (74.6)	103 (60.2)	0.08
**CD, *n* (%)**	14 (23.7)	64 (37.4)	0.11
**IBD-U, *n* (%)**	1 (1.7)	5 (2.9)	0.61
**Age at diagnosis of IBD (years), median (range)**	27 (4-69)	24 (7-78)	0.77
**Age at biopsy (years), median (range)**	36 (18-80)	37 (18-82)	0.55
**Disease duration at biopsy (years), median (range)**	8 (0-51)	8 (0-44)	0.89
**History of CMV reactivation, *n* (%)**	9 (15.3)	23 (13.5)	0.73
**History of bowel resection(s), *n* (%)**	10 (16.9)	48 (28.1)	0.09
**In-patient stay at time of biopsy, *n* (%)**	31 (52.5)	43 (25.1)	**0.001**
**Duration of in-patient stay (days), mean** **±** **SD**	19 ± 24	12 ± 7	**0.001**
**Medical treatment**			
**Anti-TNFα treatment at biopsy, *n* (%)**	14 (23.7)	37 (21.6)	0.76
**Anti-interleukin 12/23 treatment at biopsy, *n* (%)**	10 (16.9)	26 (15.2)	0.76
**Anti-integrin treatment at biopsy, *n* (%)**	5 (8.5)	19 (11.1)	0.56
**Calcineurin inhibitor treatment at biopsy, *n* (%)**	9 (15.3)	12 (7.0)	0.06
**Azathioprine/6-Mercatopurine treatment at biopsy, *n* (%)**	7 (11.9)	19 (11.1)	0.89
**Methotrexate treatment at biopsy, *n* (%)**	0 (0)	1 (0.6)	0.56
**JAK-inhibitor treatment at biopsy, *n* (%)**	2 (3.4)	15 (8.8)	0.17
**Steroid treatment (intravenous) at biopsy, *n* (%)**	12 (20.3)	7 (4.1)	**0.004**
**Steroid treatment (oral) at biopsy, *n* (%)**	22 (37.3)	47 (27.5)	0.16
**Combined treatment at biopsy, *n* (%)**	29 (49.2)	67 (39.2)	0.19
**Montreal classification of UC and IBD-U:**			
**E1**	1 (1.7)	5 (2.9)	0.15
**E2**	10 (16.9)	31 (18.1)	0.84
**E3**	33 (55.9)	58 (33.9)	**0.003**
**Montreal classification of CD:**			
**L1**	4 (6.8)	10 (5.8)	0.8
**L2**	5 (8.5)	18 (10.5)	0.58
**L3**	4 (6.8)	38 (22.2)	**0.002**
**L4**	0 (0)	9 (5.3)	0.31
**B1**	8 (13.6)	22 (12.9)	0.89
**B2**	2 (3.4)	26 (15.2)	0.053
**B3**	4 (6.8)	25 (14.6)	0.12
**Perianal disease**	3 (5.1)	22 (12.9)	0.1
**Clinical disease activity**			
**SCCAI at biopsy, median (range) (UC and IBD-U, *n* = 137)**	8 (0-14)	5.5 (0-14)	**0.02**
**HBI at biopsy, median (range) (CD, *n* = 76)**	9 (0-36)	6 (0-21)	0.08
**Laboratory parameters**			
**CRP (mg/L), mean** **±** **SD**	31 ± 42	20 ± 35	**0.04**
**Calprotectin (µg/g), mean** **±** **SD**	1325 ± 633	850 ± 702	0.17
**Endoscopic disease activity**			
**Mayo score, median (range) (UC)**	2 (0-3)	2 (0-3)	**0.003**
**SES-CD score, median (range) (CD)**	8 (6-20)	7 (0-27)	0.17
**Endoscopic findings**			
**Presence of ulcers, *n* (%)**	35 (59.3)	78 (45.6)	0.08
**Deep ulcers, *n* (%)**	14 (23.7)	13 (7.6)	**0.001**
**Histological findings**			
**Acute inflammation, *n* (%)**	53 (89.8)	128 (74.9)	**0.03**
**Chronic inflammation, *n* (%)**	45 (76.3)	125 (73.1)	0.83
**Simultaneous viral DNA detection**			
**Positive CMV PCR**	3 (5.1)		
**Positive HHV-6 PCR**	6 (10.2)		

Abbreviations: CD: Crohn’s disease; CMV: cytomegalovirus; EBV: Epstein-Barr virus; HHV-6: human herpesvirus-6; IBD-U: IBD unclassified; HBI: Harvey-Bradshaw Index; IBD: inflammatory bowel disease; JAK: Janus kinase; PCR, polymerase chain reaction; SCCAI simple clinical colitis activity index; SES-CD: simple endoscopic score for Crohn′s disease; TNFα: tumor necrosis factor alpha; UC: ulcerative colitis.

Bold values indicate significant *P*-values (<0.05).

### Human herpesvirus 6

HHV-6 DNA was found in 20 patients (*n* = 20/230; 8.7%). Of those 20 patients (9 male, median age 29 years), UC was diagnosed in 14 patients, and CD in 6 patients. In brief, patients with positive HHV-6 PCR test results were more frequently on azathioprine or 6-mercaptopurine treatment (*P *= 0.006) or oral steroid treatment (*P *= 0.04) compared to patients with HHV-6 PCR negative test results.

Among HHV-6 positive patients, 6 (*n* = 6/20; 30%) also tested positive for EBV, while no patient had simultaneous positive CMV PCR test results. Detailed characteristics of the patients with positive and negative HHV-6 PCR are presented in Table 4.

**Table 4:** Comparison of characteristics between the subgroups of patients with positive HHV-6 PCR and negative HHV-6 PCR.

	HHV-6 PCR-positive	HHV-6 PCR -egative	*p*-value
	*n* = 20/230	*n* = 210/230	
**Variable**			
**Male, *n* (%)**	9 (45)	114 (54.3)	0.43
**IBD specification, *n* (%)**			
**UC, *n* (%)**	14 (70)	132 (62.9)	0.53
**CD, *n* (%)**	6 (30)	72 (34.3)	0.7
**IBD-U, *n* (%)**	0 (0)	6 (2.8)	0.44
**Age at diagnosis of IBD (years), median (range)**	23 (16-60)	26 (4-78)	0.39
**Age at biopsy (years), median (range)**	29 (19-60)	37 (18-82)	**0.03**
**Disease duration at biopsy (years), median (range)**	3.5 (0-21)	8 (0-51)	**0.03**
**History of CMV reactivation, *n* (%)**	8 (20)	24 (11.4)	**0.001**
**History of bowel resection(s), *n* (%)**	3 (15)	55 (26.2)	0.27
**In-patient stay at time of biopsy, *n* (%)**	8 (40.0)	66 (31.4)	0.44
**Duration of in-patient stay (days), mean** **±** **SD**	19 ± 14	14 ± 17	0.68
**Medical treatment**			
**Anti-TNFα treatment at biopsy, *n* (%)**	4 (20)	47 (22.4)	0.8
**Anti-interleukin 12/23 treatment at biopsy, *n* (%)**	3 (15)	33 (15.7)	0.93
**Anti-integrin treatment at biopsy, *n* (%)**	0 (0)	24 (11.4)	0.11
**Calcineurin inhibitor treatment at biopsy, *n* (%)**	0 (0)	21 (10)	0.14
**Azathioprine/6-Mercatopurine treatment at biopsy, *n* (%)**	6 (30)	20 (9.5)	**0.006**
**Methotrexate treatment at biopsy, *n* (%)**	0 (0)	1 (0.5)	0.76
**JAK-inhibitor treatment at biopsy, *n* (%)**	2 (10)	15 (7.1)	0.65
**Steroid treatment (intravenous) at biopsy, *n* (%)**	3 (15)	16 (7.6)	0.26
**Steroid treatment (oral) at biopsy, *n* (%)**	10 (50)	60 (28.6)	**0.04**
**Combined treatment at biopsy, *n* (%)**	10 (50)	86 (40.9)	0.44
**Montreal classification of UC and IBD-U:**			
**E1**	0 (0)	6 (2.9)	0.44
**E2**	8 (40)	33 (25.7)	**0.007**
**E3**	6 (30)	85 (40.5)	0.38
**Montreal classification of CD:**			
**L1**	1 (5)	13 (6.2)	0.83
**L2**	3 (15)	20 (9.5)	0.44
**L3**	2 (10)	40 (19.1)	0.32
**L4**	0 (0)	9 (4.3)	0.35
**B1**	1 (5)	29 (13.8)	0.27
**B2**	2 (10)	26 (12.4)	0.76
**B3**	3 (15)	26 (12.4)	0.74
**Perianal disease**	5 (25)	21 (10)	0.17
**Clinical disease activity**			
**SCCAI at biopsy, median (range) (UC and IBD-U, *n* = 137)**	8 (2-13)	6 (0-14)	0.34
**HBI at biopsy, median (range) (CD, *n* = 76)**	12 (0-20)	6 (0-36)	0.26
**Laboratory parameters**			
**CRP (mg/L), mean** **±** **SD**	21 ± 33	23 ± 38	0.77
**Calprotectin (µg/g), mean** **±** **SD**	995 ± 795	977 ± 718	0.58
**Endoscopic disease activity**			
**Mayo score, median (range) (UC)**	2 (1-3)	2 (0-3)	0.88
**SES-CD score, median (range) (CD)**	4 (2-6)	7.5 (0-27)	0.3
**Endoscopic findings**			
**Presence of ulcers, *n* (%)**	12 (60.0)	101 (48.1)	0.22
**Deep ulcers, *n* (%)**	3 (15.0)	24 (11.4)	0.58
**Histological findings**			
**Acute inflammation, *n* (%)**	16 (80.0)	165 (78.6)	0.99
**Chronic inflammation, *n* (%)**	16 (80.0)	154 (73.3)	0.6
**Simultaneous viral DNA detection**			
**Positive CMV PCR**	0 (0)		
**Positive EBV PCR**	6 (30)		

Abbreviations: CD: Crohn’s disease; CMV: cytomegalovirus; EBV: Epstein-Barr virus; HHV-6: human herpesvirus-6; IBD-U: IBD unclassified; HBI: Harvey-Bradshaw Index; IBD: inflammatory bowel disease; JAK: Janus kinase; PCR, polymerase chain reaction; SCCAI simple clinical colitis activity index; SES-CD: simple endoscopic score for Crohn′s disease; TNFα: tumor necrosis factor alpha; UC: ulcerative colitis.

Bold values indicate significant *P*-values (<0.05).

### Factors associated with CMV, EBV, and HHV-6 DNA positivity in inflammatory bowel disease

Univariate regression analysis identified lower disease activity (SSCAI: OR 0.74, *P *= 0.02; Mayo score: OR 0.39, *P *= 0.01), and treatment with JAK-inhibitors (OR 1.16, *P *= 0.03) as risk factors for CMV DNA positivity, while multivariate analysis could not confirm lower disease activity or JAK inhibitor treatment as independent risk factors for CMV DNA positivity.

Higher disease activity (SSCAI: OR 1.12, *P *= 0.02; HBI: OR 1.11, *P *= 0.01; Mayo score: OR 2.1, *P *= 0.004), and treatment with steroids (OR 1.13, *P *= 0.001) were identified as risk factors for EBV DNA positivity by univariate regression analysis (Table 5). Multivariate analysis revealed the higher disease activity (HBI: OR 1.11, *P *= 0.01; Mayo: OR 1.96, *P *= 0.02) and steroid treatment (OR 3.8, *P *= 0.03) as independent risk factors for EBV DNA positivity.

**Table 5:** Risk factor analyses for EBV DNA positivity in IBD patients.

Risk factor	Univariate		Multivariate	
	OR (95% CI)	*P*-value	OR (95% CI)	*P*-value
**Sex**	0.73 (0.39-1.32)	0.29		
**Age at biopsy**	1.00 (0.99-1.03)	0.39		
**Age at diagnosis of IBD**	1.00 (0.98-1.02)	0.98		
**Disease duration at biopsy**	1.01 (0.98-1.04)	0.46		
**History of CMV reactivation**	0.86 (0.37-1.99)	0.73		
**History of bowel resection(s)**	1.93 (0.9-4.11)	0.09		
**Type of IBD**				
**UC**	0.48 (0.05-4.22)	0.51		
**CD**	0.84 (0.09-7.73	0.88		
**IBD-U**	1.75 (0.2-15.27)	0.61		
**Clinical disease activity**				
**SSCAI at biopsy**	1.12 (1.02-1.24)	**0.02**	1.04 (0.93-1.17)	0.5
**HBI at biopsy**	1.11 (1.02-1.21)	**0.01**	1.11 (1.02-1.21)	**0.01**
**Endoscopic disease activity**				
**Mayo score**	2.1 (1.28-3.44)	**0.004**	1.96 (1.12-3.43)	**0.02**
**SES-CD score**	1.06 (0.95-1.19)	0.31		
**Medical treatment**				
**Anti-TNFα treatment**	0.96 (0.45-2.05)	0.91		
**Anti-interleukin 12/23 treatment**	0.86 (0.36-2.05)	0.74		
**Anti-integrin treatment**	1.45 (0.49-4.31)	0.51		
**Calcineurin inhibitor treatment**	0.43 (0.17-1.11)	0.08		
**Azathioprine/6-Mercatopurine treatment**	0.97 (0.37-2.49)	0.94		
**Methotrexate treatment**				
**JAK-inhibitor treatment**	2.76 (0.61-12.44)	0.19		
**Steroid treatment (intravenous)**	1.13 (1.05-1.37)	**0.001**	3.8 (1.1-13.1)	**0.034**
**Steroid treatment (oral)**	0.64 (0.34-1.2)	0.17		
**Combined treatment**	0.67 (0.37-1.22)	0.19		

CD: Crohn’s disease; CI, confidence interval; EBV: Epstein-Barr virus; IBD-U: IBD unclassified; HBI: Harvey-Bradshaw Index; IBD: inflammatory bowel disease; JAK: Janus kinase; OR: odds ratio; PCR, polymerase chain reaction; SCCAI simple clinical colitis activity index; SES-CD: Simple Endoscopic Score for Crohn′s Disease; TNFα: Tumor necrosis factor alpha; UC: ulcerative colitis. Bold values indicate significant *P*-values (<0.05).

As shown in Table 6, the univariate analysis identified younger age at biopsy (OR 0.96, *P *= 0.03), disease duration (OR 0.93, *P *= 0.04), history of CMV reactivation (OR 1.2, *P *= 0.002) and treatment with azathioprine/6-mercatopurine (OR 2.25, *P *= 0.01) or steroids (OR 1.39, *P *= 0.05) as independent risk factors for HHV-6 DNA positivity. History of CMV reactivation (OR 4.7, *P *= 0.02), and treatment with azathioprine/6-mercatopurine (OR 7.58, *P *= 0.005) or steroids (OR 4.26, *P *= 0.02) were confirmed as independent risk factors by multivariate analysis. Risk factor analyses for CMV DNA positivity are shown in Table 7.

**Table 6:** Risk factor analyses for HHV-6 DNA positivity in IBD patients.

Risk factor	Univariate		Multivariate	
	OR (95% CI)	*P*-value	OR (95% CI)	*P*-value
**Sex**	1.45 (0.58-3.65)	0.43		
**Age at biopsy**	0.96 (0.93-0.99)	**0.03**	0.96 (0.92-1.00)	0.09
**Age at diagnosis of IBD**	0.99 (0.95-1.01)	0.38		
**Disease duration at biopsy**	0.93 (0.87-0.99)	**0.04**	0.99 (0.91-1.08)	0.83
**History of CMV reactivation**	1.20 (1.08-4.54)	**0.002**	4.7 (1.24-17.85)	**0.02**
**History of bowel resection(s)**	2.02 (0.57-7.17)	0.27		
**Type of IBD**				
**UC**	0.72 (0.27-1.97)	0.53		
**CD**	1.2 (0.45-3.3)	0.69		
**IBD-U**				
**Clinical disease activity**				
**SSCAI at biopsy**	1.08 (0.93-1.25)	0.33		
**HBI at biopsy**	1.05 (0.95-1.17)	0.31		
**Endoscopic disease activity**				
**Mayo score**	1.00 (0.54-1.87)	1.00		
**SES-CD score**	0.85 (0.61-1.18)	0.33		
**Medical treatment**				
**Anti-TNFα treatment**	1.16 (0.37-3.64)	0.79		
**Anti-interleukin 12/23 treatment**	1.06 (0.29-3.83)	0.93		
**Anti-integrin treatment**		0.99		
**Calcineurin inhibitor treatment**		0.99		
**Azathioprine/6-Mercatopurine treatment**	2.25 (1.08-5.71)	**0.01**	7.58 (1.82-31.6)	**0.005**
**Methotrexate treatment**				
**JAK-inhibitor treatment**	0.69 (0.15-3.29)	0.65		
**Steroid treatment (intravenous)**	0.47 (0.12-1.77)	0.26		
**Steroid treatment (oral)**	1.39 (1.16-3.99)	**0.05**	4.26 (1.23-14.69)	**0.02**
**Combined treatment**	0.70 (0.28-1.75)	0.45		

Abbreviations: CD: Crohn’s disease; CI, confidence interval; IBD-U: IBD unclassified; HBI: Harvey-Bradshaw Index; HHV-6: human herpesvirus-6; IBD: inflammatory bowel disease; JAK: Janus kinase; OR: odds ratio; PCR, polymerase chain reaction; SCCAI simple clinical colitis activity index; SES-CD: simple endoscopic score for Crohn′s disease; TNFα: tumor necrosis factor alpha; UC: ulcerative colitis.

Bold values indicate significant *P*-values (<0.05).

**Table 7:** Risk factor analyses for CMV DNA positivity in IBD patients.

Risk factor	Univariate		Multivariate	
	OR (95% CI)	*P*-value	OR (95% CI)	*P*-value
**Sex**	0.85 (0.29-2.54)	0.78		
**Age at biopsy**	1.01 (0.98-1.04)	0.52		
**Age at diagnosis of IBD**	1.00 (0.97-1.04)	0.99		
**Disease duration at biopsy**	1.02 (0.98-1.07)	0.28		
**History of CMV reactivation**	1.01 (0.21-4.75)	0.99		
**History of bowel resection(s)**	0.59 (0.19-1.84)	0.36		
**Type of IBD**				
**UC**	2.72 (0.29-25.58)	0.38		
**CD**	5.00 (0.44-57.22)	0.19		
**IBD-U**	0.31 (0.03-2.83)	0.29		
**Clinical disease activity**				
**SSCAI at biopsy**	0.74 (0.57-0.95)	**0.02**	0.8 (0.61-1.04)	0.09
**HBI at biopsy**	0.92 (0.77-1.1)	0.36		
**Endoscopic disease activity**				
**Mayo score**	0.39 (0.19-0.81)	**0.01**	0.44 (0.18-1.09)	0.08
**SES-CD score**	0.89 (0.61-1.32)	0.57		
**Medical treatment**				
**Anti-TNFα treatment**	0.54 (0.11-2.6)	0.44		
**Anti-interleukin 12/23 treatment**	0.43 (0.09-2.09)	0.29		
**Anti-integrin treatment**	0.35 (0.06-2.13)	0.25		
**Calcineurin inhibitor treatment**	0.91 (0.10-7.92)	0.93		
**Azathioprine/6-Mercatopurine treatment**	0.61 (0.12-3.21)	0.56		
**Methotrexate treatment**				
**JAK-inhibitor treatment**	1.16 (1.03-1.8)	**0.03**	5.33 (0.94-30.14)	0.06
**Steroid treatment (intravenous)**	1.1 (0.13-9.18)	0.93		
**Steroid treatment (oral)**	0.77 (0.24-2.42)	0.66		
**Combined treatment**	0.71 (0.24-2.09)	0.53		

CD: Crohn’s disease; CI, confidence interval; CMV: Cytomegalovirus; IBD-U: IBD unclassified; HBI: Harvey-Bradshaw Index; IBD: inflammatory bowel disease; JAK: Janus kinase; OR: odds ratio; PCR, polymerase chain reaction; SCCAI simple clinical colitis activity index; SES-CD: Simple Endoscopic Score for Crohn′s Disease; TNFα: Tumor necrosis factor alpha; UC: ulcerative colitis.

Bold values indicate significant *P*-values (<0.05).

## Discussion

So far, the pathogenetic and clinical relevance of the herpesviruses EBV and HHV-6 and to a lesser extent, of CMV in IBDs remain elusive. Although there is a growing interest in opportunistic viral infections in IBD, several issues remain unsolved. Therefore, this study evaluated the prevalence of CMV, EBV, and HHV-6 DNA in intestinal mucosal tissue samples from patients with IBD, and aimed to investigate potential associations between the clinical course of the disease, disease activity, and endoscopic/histological findings. To the best of our knowledge, this is the first study including HHV-6 in the analysis and comparing 3 herpesviruses in a comprehensive approach. We found that the prevalence of EBV in the intestinal mucosa within the IBD study cohort was 25.7%, which is the highest prevalence of all investigated herpesviruses. Moreover, the prevalence of EBV seems to correlate with disease severity and activity. Patients with EBV-positive PCR test results were hospitalized more often and had to stay longer in the hospital. This is in line with other study results showing EBV as the most prevalent infection, with reported rates ranging from 16% to 79.4%.^6^^,^^15^^,^^22^^,^^23^ Other studies suggest that EBV infection might be associated with worse clinical outcome, exacerbation of symptoms, and refractory IBD.^6^^,^^15^^,^^22^^,^^24^ In agreement with these findings, EBV-positive IBD patients were more often on systemic steroid treatment. Interestingly, treatment with new biological agents was not associated with the detection of EBV DNA in the patients of our study. This observation is of great clinical relevance; as most scientific publications so far have focused on CMV infections in IBD. Furthermore, it is of note that the treatment strategy of IBD has changed over the last decades to an earlier and more aggressive therapy intended to achieve mucosal healing and to prevent disease progression.^1^ Hence, a substantial number of patients are on immunosuppressive medications or biologicals, which may lead to an increased risk of gastrointestinal infections. Regarding EBV, more than 90% of adults worldwide are seropositive for EBV, as EBV infection is usually established in childhood or adolescence, with lifelong persistence in the latent phase within resting memory B cells.^25^^,^^26^ Although different studies suggest that age, irregular ulceration, and therapy with steroids or immunosuppressant’s might be associated with EBV infection in IBD patients,^6^^,^^15^^,^^27^^,^^28^ several studies could not show any influence on the EBV load by treatment with azathioprine or anti-TNFα-antibodies in CD patients.^29^^,^^30^ In addition, the prevalence of EBV DNA was found to be higher in UC patients compared to CD patients, suggesting that UC might be considered a risk factor for EBV PCR positivity. This observation could be explained by the different characteristics of the TH-2 pattern and elevated serum levels of soluble CD30 protein in patients with UC, leading to increased frequencies of a high EBV load and productive infection in active UC patients compared to CD patients.^28^ However, it must be considered that in general practice, patients with UC flares might receive more frequent viral testing compared to patients with CD—a potential source of bias in the data.

Another study revealed that the existence of mucosal inflammation and the use of corticosteroids, cyclosporine A, or tacrolimus were significant risk factors for EBV infection.^31^ One study found a close association between the use of biologic agents and increased EBV detection in intestinal mucosa, but found no association between the development of viral end-organ disease and the use of immunosuppressants, the duration of therapies and underlying disease, age, gender, smoking habits, or body mass index.^6^ These results differ from our study results, indicating no increased risk for EBV DNA positivity attributed to treatment with new biological agents. Notably, a considerable proportion of our patients (42%) were on combined immunosuppressive therapy at the time of biopsy, which might predispose the patient to viral reactivation, making it difficult to attribute the risk to a single drug. Another important factor could be the time of biopsy in relation to disease activity and therapy modifications. Due to the retrospective design of this study, biopsies were taken at different stages of the IBD flare, with the patient possibly having already changed treatments or started antiviral therapy, which could influence virus detection. On the other hand, a DNA-positive mucosal biopsy does not necessarily equate to an active virus infection, but might depend more on technical factors or the severity of the mucosal inflammation. More than 90% of adults are EBV carriers with a lifelong EBV persistence in B cells of the mucosa,^25^^,^^26^ while HHV-6 may even be integrated into human chromosomes in some individuals.^32^ Especially for EBV and HHV-6, there are no clear established clinical definitions for a viral colitis. Keeping this in mind, the sole detection of a viral DNA without viral load quantification or evidence of viral protein expression (eg, immunohistochemistry or in situ hybridization) seems to be insufficient to diagnose viral colitis. In all, the associations observed in this study should be interpreted with caution regarding the above-mentioned confounding factors (eg, immunosuppressive therapy, disease severity, and the omnipresence of the viruses). The described correlations are neither inevitably specific nor causal. It remains unclear if EBV triggers IBD activity or is only an epiphenomenon of severe inflammation. Notably, patients with more aggressive IBD courses are more likely to be on severe immunosuppressive treatment and more likely to exhibit a disruption of the mucosal barrier, both of which might facilitate a reactivation of latent viruses like EBV. Our study results show a correlation between EBV PCR positivity and IBD severity, but causality cannot be proven. Further controlled prospective studies are needed to distinguish between cause and effect.

The role of HHV-6 in IBD patients is elusive and data on HHV-6-associated colitis are rare.^33^^,^^34^ One study showed a prevalence of HHV-6 in 9.2% of IBD patients. However, there was no difference among UC patients, CD patients, and healthy controls.^31^ Our study results suggested some associations between HHV-6 PCR positivity and azathioprine/6-mercaptopurine treatment and steroid treatment. Similar to the reported prevalence in the study mentioned above, the prevalence of HHV-6 in our study cohort was low (8.7%), indicating that further studies with larger cohorts are warranted to investigate the detailed role of HHV-6 in this context.

Regarding CMV, the use of corticosteroids, azathioprine or 6-mercaptopurine, and TNFα-antibodies were identified as risk factors for viral reactivation, with combined treatment increasing the risk even more.^35^^,^^36^ Nevertheless, TNFα-antibodies seem not to affect the incidence of viral reactivation in the short-term, but mostly in long-term treatment.^37^^,^^38^ On the other hand, several studies identified systemic use of corticosteroids as a risk factor for both CMV and EBV colitis in transplant patients,^39^^,^^40^ which might be attributed to the tendency of steroids to increase viral protein production.^40^ Our study results for CMV could only partially confirm these data, and surprisingly, the CMV-positive patients in our cohort did not show higher clinical or endoscopic severity scores. This finding seems paradoxical, as the presence of CMV colitis is usually expected in the more severe cases. Hence, limited by the small number of patients included and the retrospective study design, these study results must be interpreted with caution. Statistical analyses might be limited in this context, especially since multivariate analyses could not confirm these observations in this study. Moreover, patients would have received antiviral therapy or intensified care once CMV was detected, thereby distorting short-term outcome parameters. In fact, all CMV-positive patients in this study cohort were treated with antiviral therapy. Colectomy was recommended to 1 patient due to therapy-refractory colitis, but this patient refused the operation until the last follow-up. Moreover, disease activity parameters and endoscopy scores were calculated retrospectively from the patient records which might also distort results. Another limiting factor is the median follow-up time of only 12 months in this study, which is perhaps too short to evaluate long-term prognosis. However, the relationship between CMV positivity and long-term prognosis represents a highly important and intriguing aspect that warrants further investigation in future studies with long-term follow-up. Altogether, the role of CMV as a trigger or epiphenomenon in IBD flares remains debatable, and our study results cannot prove causality.

A previous study identified overlapping CMV and EBV or HHV-6 infection as a significant and independent prognostic factor for subsequent colorectal resection in patients with UC.^31^ Among the EBV-positive patients in this study, 6 patients tested concurrently positive for HHV-6 DNA, and 3 patients tested positive for CMV DNA. Five of these 9 patients were diagnosed with UC. None of these patients received subsequent colorectal resection or other IBD-related operations during a median follow-up of 12 months. This discrepancy of the study results could be explained by the significantly shorter follow-up time of 12 months in our study, compared to the median follow-up time of 78 months reported in the other study. Furthermore, our patient number remains limited. Further studies with longer follow-up periods and larger patient cohorts must be conducted to identify the impact of overlapping CMV and EBV or HHV-6 infection in IBD.

While there is an established therapy for patients with intestinal CMV reactivation using (val)ganciclovir,^7^ effective treatment strategies for EBV and HHV are lacking, and there is no consensus on the necessity of therapy for the optimal therapy itself. Despite the withdrawal or reduction of ongoing immunosuppressive therapy, most patients with EBV colitis undergo colectomy.^6^ Antiviral therapies are still controversial, and the application of rituximab—a CD20-antibody which is usually applied for the treatment of malignant hematological diseases, lymphomas, and autoimmune diseases—might serve as rescue therapy for severe cases. Due to the rapid depletion of B lymphocytes by rituximab, the host of EBV is destroyed.^6^^,^^41^ Nevertheless, there is still no effective therapy available to date, and 1 might ask whether an antiviral treatment would be necessary if the detection of the virus represents only an epiphenomenon of IBD flares. Hence, for a more precise determination of clinical relevance, it is crucial to clarify whether the viruses are triggers or bystanders.

This study has several limitations, the most important one of which is its retrospective design. Regarding our results—especially concerning CMV and HHV-6—the statistical analysis and the calculated *P*-values should be interpreted with caution, as the number of affected patients was small. Furthermore, there might be a potential selection bias, because patients with more severe or refractory disease were more likely to undergo a biopsy for suspected CMV reactivation, although IBD patients in our center would also be routinely tested for CMV in their annual follow-up endoscopy. Hence, our study results may overestimate the prevalence of viral DNA compared to that of the broader IBD population, as our study cohort represents the more severe cases of a tertiary referral center for IBD. Another limitation is the absence of a control group, which makes it difficult to determine whether the observed differences are due to underlying factors or random variation rather than true associations. Moreover, the retrospective study design aggravates a standardization of data (eg, consistent biopsy protocol). Another limitation for external validity is the monocentric design of this study reflecting local practices of 1 center, while patient management and testing thresholds at other centers might differ. This single-center design might limit the diversity of the patient population. Altogether, the data of this study have to be interpreted with caution and generalizability must be limited. As this is a descriptive study showing associations, causality cannot be proven. Due to the study design, mainly cross-sectional associations at the time of biopsy and no longitudinal results were recorded. Furthermore, a key limitation is the qualitative viral PCR detection without quantitative assessment or histopathology. Without quantification of the viral load, it is difficult to distinguish between an active infection—which is usually indicated by high viral load—and a latent or clinically insignificant course, with low viral loads. There was also no correlation of the PCR results with histopathology or immunohistochemistry for these viruses in this study, as recommended by the ECCO guidelines for CMV colitis.^7^ This makes it difficult to confirm that detection of viral DNA represents clinically significant colitis caused by the viruses, because qualitative PCR cannot distinguish latent and/or integrated virus from active replication. Furthermore, the possibility of sampling error with false negative results during the biopsies and virologic analysis must also be considered. Due to the small number of virus PCR-positive patients, statistical analysis is limited for multiple comparisons and adjusted analyses.

Regardless of these limitations, our observations might be relevant for the diagnosis and therapy of IBD. Further prospective controlled multi-center studies are warranted to confirm our results in general and to distinguish between correlation and causation.

## Conclusion

In conclusion, we have shown that the prevalence of EBV is high in the intestinal mucosa of patients with IBD, and correlates with disease severity and activity, although causality cannot be proven. HHV-6 PCR positivity and EBV PCR positivity are associated with immunosuppressive therapies.

## Data Availability

The datasets used and/or analyzed during the current study are available from the corresponding author on reasonable request.

## References

[1] Hanauer SB , Kornbluth AA, Messick J, Rubin DT, Sandborn WJ, Sands BE. Clinical scenarios in IBD: optimizing the use of conventional and biologic agents. Inflamm Bowel Dis. 2010;16 Suppl 1:S1-S11. 10.1002/ibd.21529 21104734

[2] Powell RD , Warner NE, Levine RS, Kirsner JB. Cytomegalic inclusion disease and ulcerative colitis; report of a case in a young adult. Am J Med. 1961;30:334-340. 10.1016/0002-9343(61)90105-x 13737621

[3] Kandiel A , Lashner B. Cytomegalovirus colitis complicating inflammatory bowel disease. Am J Gastroenterol. 2006;101:2857-2865. 10.1111/j.1572-0241.2006.00869.x 17026558

[4] Roblin X , Pillet S, Oussalah A, et al. Cytomegalovirus load in inflamed intestinal tissue is predictive of resistance to immunosuppressive therapy in ulcerative colitis. Am J Gastroenterol. 2011;106:2001-2008. 10.1038/ajg.2011.202 21788989

[5] Hamlin PJ , Shah MN, Scott N, Wyatt JI, Howdle PD. Systemic cytomegalovirus infection complicating ulcerative colitis: a case report and review of the literature. Postgrad Med J. 2004;80:233-235. 10.1136/pgmj.2003.007385 PMC1742981 15082847

[6] Ciccocioppo R , Racca F, Paolucci S, et al. Human cytomegalovirus and Epstein-Barr virus infection in inflammatory bowel disease: need for mucosal viral load measurement. World J Gastroenterol. 2015;21:1915-1926. 10.3748/wjg.v21.i6.1915 PMC4323471 25684960

[7] Kucharzik T , Ellul P, Greuter T, et al. ECCO Guidelines on the Prevention, Diagnosis, and Management of Infections in Inflammatory Bowel Disease. J Crohns Colitis. 2021;15:879-913. 10.1093/ecco-jcc/jjab052 33730753

[8] Nahar S , Iraha A, Hokama A, et al. Evaluation of a multiplex PCR assay for detection of cytomegalovirus in stool samples from patients with ulcerative colitis. World J Gastroenterol. 2015;21:12667-12675. 10.3748/wjg.v21.i44.12667 PMC4658622 26640344

[9] Wakefield AJ , Fox JD, Sawyerr AM, et al. Detection of herpesvirus DNA in the large intestine of patients with ulcerative colitis and Crohn’s disease using the nested polymerase chain reaction. J Med Virol. 1992;38:183-190. 10.1002/jmv.1890380306 1287131

[10] Maher MM , Nassar MI. Acute cytomegalovirus infection is a risk factor in refractory and complicated inflammatory bowel disease. Dig Dis Sci. 2009;54:2456-2462. 10.1007/s10620-008-0639-6 19093204

[11] Leveque N , Brixi-Benmansour H, Reig T, et al. Low frequency of cytomegalovirus infection during exacerbations of inflammatory bowel diseases. J Med Virol. 2010;82:1694-1700. 10.1002/jmv.21877 20827767

[12] Cottone M , Pietrosi G, Martorana G, et al. Prevalence of cytomegalovirus infection in severe refractory ulcerative and Crohn’s colitis. Am J Gastroenterol. 2001;96:773-775. 10.1111/j.1572-0241.2001.03620.x 11280549

[13] Bertalot G , Villanacci V, Gramegna M, et al. Evidence of Epstein-Barr virus infection in ulcerative colitis. Dig Liver Dis. 2001;33:551-558. 10.1016/s1590-8658(01)80106-7 11816543

[14] Yanai H , Shimizu N, Nagasaki S, Mitani N, Okita K. Epstein-Barr virus infection of the Colon with inflammatory bowel disease. Am J Gastroenterol. 1999;94:1582-1586. 10.1111/j.1572-0241.1999.01148.x 10364028

[15] Zhang H , Zhao S, Cao Z. Impact of Epstein-Barr virus infection in patients with inflammatory bowel disease. Front Immunol. 2022;13:1001055. 10.3389/fimmu.2022.1001055 PMC9651941 36389673

[16] Maaser C , Sturm A, Vavricka SR, et al. ECCO-ESGAR Guideline for Diagnostic Assessment in IBD Part 1: Initial diagnosis, monitoring of known IBD, detection of complications. J Crohns Colitis. 2019;13:144-164. 10.1093/ecco-jcc/jjy113 30137275

[17] Satsangi J , Silverberg MS, Vermeire S, Colombel JF. The Montreal classification of inflammatory bowel disease: controversies, consensus, and implications. Gut. 2006;55:749-753. 10.1136/gut.2005.082909 PMC1856208 16698746

[18] Walmsley RS , Ayres RC, Pounder RE, Allan RN. A simple clinical colitis activity index. Gut. 1998;43:29-32. 10.1136/gut.43.1.29 PMC1727189 9771402

[19] Harvey RF , Bradshaw JM. A simple index of Crohn’s-disease activity. Lancet. 1980;1:514. 10.1016/s0140-6736(80)92767-1 6102236

[20] Schroeder KW , Tremaine WJ, Ilstrup DM. Coated oral 5-aminosalicylic acid therapy for mildly to moderately active ulcerative colitis. A randomized study. N Engl J Med. 1987;317:1625-1629. 10.1056/NEJM198712243172603 3317057

[21] Daperno M , D’Haens G, Van Assche G, et al. Development and validation of a new, simplified endoscopic activity score for Crohn’s disease: the SES-CD. Gastrointest Endosc. 2004;60:505-512. 10.1016/s0016-5107(04)01878-4 15472670

[22] Dimitroulia E , Pitiriga VC, Piperaki ET, Spanakis NE, Tsakris A. Inflammatory bowel disease exacerbation associated with Epstein-Barr virus infection. Dis Colon Rectum. 2013;56:322-327. 10.1097/DCR.0b013e31827cd02c 23392146

[23] Ryan JL , Shen YJ, Morgan DR, et al. Epstein-Barr virus infection is common in inflamed gastrointestinal mucosa. Dig Dis Sci. 2012;57:1887-1898. 10.1007/s10620-012-2116-5 PMC3535492 22410851

[24] Pezhouh MK , Miller JA, Sharma R, et al. Refractory inflammatory bowel disease: is there a role for Epstein-Barr virus? A case-controlled study using highly sensitive Epstein-Barr virus-encoded small RNA1 in situ hybridization. Hum Pathol. 2018;82:187-192. 10.1016/j.humpath.2018.08.001 30120969

[25] Hosomi S , Nishida Yu, Fujiwara Y. The impact of human herpesviruses in clinical practice of inflammatory bowel disease in the era of COVID-19. *Microorganisms*. 2021;9:1870. 10.3390/microorganisms9091870 PMC8468540 34576764

[26] Cohen JI. Epstein-Barr virus infection. N Engl J Med. 2000;343:481-492. 10.1056/NEJM200008173430707 10944566

[27] Nissen LH , Nagtegaal ID, de Jong DJ, et al. Epstein-Barr virus in inflammatory bowel disease: the spectrum of intestinal lymphoproliferative disorders. J Crohns Colitis. 2015;9:398-403. 10.1093/ecco-jcc/jjv040 25740811

[28] Zhou JQ , Zeng L, Zhang Q, et al. Clinical features of Epstein-Barr virus in the intestinal mucosa and blood of patients with inflammatory bowel disease. Saudi J Gastroenterol. 2020;26:312-320. 10.4103/sjg.SJG_30_20 PMC8019136 33078719

[29] Fernandez Salazar L , Rojo S, De Lejarazu RO, Castro E, Higuera E, Gonzalez JM. No increase in Epstein-Barr virus viral load in a group of 30 asymptomatic patients with Crohn’s disease. Am J Gastroenterol. 2013;108:1933-1935. 10.1038/ajg.2013.250 24300873

[30] Reijasse D , Le Pendeven C, Cosnes J, et al. Epstein-Barr virus viral load in Crohn’s disease: effect of immunosuppressive therapy. Inflamm Bowel Dis. 2004;10:85-90. 10.1097/00054725-200403000-00004 15168806

[31] Hosomi S , Watanabe K, Nishida Y, et al. Combined infection of human herpes viruses: a risk factor for subsequent colectomy in ulcerative colitis. Inflamm Bowel Dis. 2018;24:1307-1315. 10.1093/ibd/izy005 29668948

[32] Arbuckle JH , Pantry SN, Medveczky MM, et al. Mapping the telomere integrated genome of human herpesvirus 6A and 6B. Virology. 2013;442:3-11. 10.1016/j.virol.2013.03.030 PMC3696530 23648233

[33] Lamoth F , Jayet PY, Aubert JD, et al. Case report: human herpesvirus 6 reactivation associated with colitis in a lung transplant recipient. J Med Virol. 2008;80:1804-1807. 10.1002/jmv.21268 18712834

[34] Delbridge MS , Karim MS, Shrestha BM, McKane W. Colitis in a renal transplant patient with human herpesvirus-6 infection. Transpl Infect Dis. 2006;8:226-228. 10.1111/j.1399-3062.2006.00143.x 17116137

[35] Toruner M , Loftus EV Jr., Harmsen WS, et al. Risk factors for opportunistic infections in patients with inflammatory bowel disease. Gastroenterology. 2008;134:929-936. 10.1053/j.gastro.2008.01.012 18294633

[36] Baroco AL , Oldfield EC. Gastrointestinal cytomegalovirus disease in the immunocompromised patient. Curr Gastroenterol Rep. 2008;10:409-416. 10.1007/s11894-008-0077-9 18627655

[37] Ford AC , Peyrin-Biroulet L. Opportunistic infections with anti-tumor necrosis factor-alpha therapy in inflammatory bowel disease: meta-analysis of randomized controlled trials. Am J Gastroenterol. 2013;108:1268-1276. 10.1038/ajg.2013.138 23649185

[38] Lavagna A , Bergallo M, Daperno M, et al. Infliximab and the risk of latent viruses reactivation in active Crohn’s disease. Inflamm Bowel Dis. 2007;13:896-902. 10.1002/ibd.20131 17345605

[39] Nebbia G , Mattes FM, Sabin CA, et al. Differential effects of prednisolone and azathioprine on the development of human cytomegalovirus replication post liver transplantation. Transplantation. 2007;84:605-610. 10.1097/01.tp.0000280555.08651.11 17876273

[40] Forbes BA , Bonville CA, Dock NL. The effects of a promoter of cell differentiation and selected hormones on human cytomegalovirus infection using an in vitro cell system. J Infect Dis. 1990;162:39-45. 10.1093/infdis/162.1.39 1693943

[41] Sankaran-Walters S , Ransibrahmanakul K, Grishina I, et al. Epstein-Barr virus replication linked to B cell proliferation in inflamed areas of colonic mucosa of patients with inflammatory bowel disease. J Clin Virol. 2011;50:31-36. 10.1016/j.jcv.2010.09.011 PMC3052968 21035384

